# Magnetic Polymeric Conduits in Biomedical Applications

**DOI:** 10.3390/mi16020174

**Published:** 2025-01-31

**Authors:** Sayan Ganguly, Shlomo Margel

**Affiliations:** 1 Department of Chemistry, University of Waterloo, Waterloo, ON N2L 3G1, Canada; sayanganguly2206@gmail.com; 2 Department of Chemistry, Bar-Ilan Institute for Nanotechnology and Advanced Materials (BINA), Bar-Ilan University, Ramat-Gan 5290002, Israel

**Keywords:** magnetic polymeric conduits, regenerative medicine, magnetic nanoparticles, tissue repair, multifunctional biomaterials

## Abstract

Magnetic polymeric conduits are developing as revolutionary materials in regenerative medicine, providing exceptional benefits in directing tissue healing, improving targeted medication administration, and facilitating remote control via external magnetic fields. The present article offers a thorough examination of current progress in the design, construction, and functionalization of these hybrid systems. The integration of magnetic nanoparticles into polymeric matrices confers distinctive features, including regulated alignment, improved cellular motility, and targeted medicinal delivery, while preserving structural integrity. Moreover, the incorporation of multifunctional attributes, such as electrical conductivity for cerebral stimulation and optical characteristics for real-time imaging, expands their range of applications. Essential studies indicate that the dimensions, morphology, surface chemistry, and composition of magnetic nanoparticles significantly affect their biocompatibility, degrading characteristics, and overall efficacy. Notwithstanding considerable advancements, issues concerning long-term biocompatibility, biodegradability, and scalability persist, in addition to the must for uniform regulatory frameworks to facilitate clinical translation. Progress in additive manufacturing and nanotechnology is overcoming these obstacles, facilitating the creation of dynamic and adaptive conduit structures designed for particular biomedical requirements. Magnetic polymeric conduits, by integrating usefulness and safety, are set to transform regenerative therapies, presenting a new avenue for customized medicine and advanced healthcare solutions.

## 1. Introduction

Magnetic polymer composites are a remarkable category of hybrid materials that integrate the structural and functional benefits of polymers with the distinctive magnetic characteristics of included magnetic particles, yielding materials with highly adjustable and flexible applications [1]. These composites are generally produced by integrating magnetic nanoparticles, such as iron oxide (Fe_3_O_4_ or γ-Fe_2_O_3_), cobalt, or nickel, into a polymer matrix that can be natural, synthetic, or a combination of both [2]. The polymer matrix imparts mechanical flexibility, chemical stability, and biocompatibility, and the magnetic nanoparticles provide characteristics including magnetic responsiveness, thermal properties, and electromagnetic shielding [3,4]. This synergy enables magnetic polymer composites to connect soft, malleable materials with rigid, magnetically active systems, rendering them suitable for diverse applications in biomedical engineering, environmental remediation, and electronics [5]. The production of these composites has advanced considerably, utilizing methods such as solution mixing, melt blending, in situ polymerization, electrospinning, and sophisticated additive manufacturing to achieve exact control over nanoparticle distribution and composite architecture [6,7]. The uniform distribution of magnetic nanoparticles throughout the polymer matrix is essential since it directly influences the composite’s performance, including its magnetic properties, mechanical strength, and thermal stability [8]. In addition to the healthcare field, magnetic polymer composites have been widely utilized in environmental applications [9]. They are utilized in water filtration systems to eliminate impurities, including heavy metals, dyes, and organic pollutants. The magnetic characteristics of the nanoparticles facilitate the straightforward separation and retrieval of composite materials post-adsorption of pollutants, rendering them a sustainable and economical alternative for water treatment. In industrial applications, these composites are employed in sensors and actuators due to their capacity to transform magnetic energy into mechanical or electrical signals [10]. Research is being conducted on flexible and lightweight magnetic polymer composites for applications in electronic devices, including flexible circuits, electromagnetic interference (EMI) shielding materials, and energy storage systems such as supercapacitors and batteries. The electrical conductivity of these composites can be improved by integrating conductive polymers or supplementary nanomaterials, such as carbon nanotubes or graphene, in conjunction with the magnetic nanoparticles [11].

In biomedical engineering, magnetic polymer composites have demonstrated revolutionary potential due to their responsiveness to external magnetic fields, a characteristic utilized for many therapeutic and diagnostic applications [12]. A notable application is in targeted medication delivery, where these composites can be designed to encapsulate pharmaceuticals and transport them to precise areas in the body under the influence of a magnetic field [13]. This method not only improves the therapeutic effectiveness of the medications but also reduces systemic side effects, as the pharmaceuticals are administered exactly where required. Magnetic polymer composites are utilized in magnetic hyperthermia, a cancer therapy method that influences the heating properties of magnetic nanoparticles in an alternating magnetic field to selectively eliminate cancer cells while preserving healthy tissues [14]. In tissue engineering, magnetic polymer composites are utilized to fabricate scaffolds that facilitate cell growth and differentiation while also offering the advantage of remote magnetic stimulation to direct the alignment of cells or tissues, especially in nerve or vascular regeneration [15,16,17]. These scaffolds are often constructed from biocompatible polymers like chitosan, alginate, or polylactic acid, which decompose over time, resulting in the formation of regenerated tissue. In surgical applications, magnetically guided instruments and implants composed of these composites facilitate minimally invasive operations, therefore decreasing recuperation durations and surgical risks [18].

Notwithstanding their myriad advantages and uses, the advancement of magnetic polymer composites presents some hurdles. Attaining a homogeneous distribution of magnetic nanoparticles inside the polymer matrix is a significant challenge, as nanoparticle aggregation can result in variations in the material’s magnetic and mechanical characteristics [19]. Surface functionalization of nanoparticles using surfactants, polymers, or other stabilizers has demonstrated efficacy in enhancing their dispersion and compatibility with the polymer matrix. A significant difficulty is guaranteeing the biocompatibility and safety of these composites, especially in biomedical applications where they directly interact with human tissues or are implanted in the body [20]. Although iron oxide nanoparticles are typically regarded as safe and sanctioned for clinical application, other magnetic substances, like cobalt and nickel, may present toxicity hazards and necessitate meticulous evaluation [21]. Furthermore, the long-term stability and degradation characteristics of these composites in diverse environments, including the human body, require further examination to ascertain their dependability and efficacy [22]. Recent developments in manufacturing techniques have mitigated some obstacles, facilitating the production of next-generation magnetic polymer composites with improved functionality. For instance, 3D printing and additive manufacturing technologies have facilitated the exact construction of intricate structures with tailored attributes, including porosity, shape, and magnetic responsiveness [23]. These methods have proven especially advantageous in biomedical applications, where there is a growing demand for patient-specific implants, scaffolds, and devices [24]. The incorporation of multifunctional characteristics into magnetic polymer composites is a burgeoning trend. Researchers are investigating methods to integrate magnetic responsiveness with other stimuli-responsive behaviors, like thermal, optical, or electrical responses, to develop “smart” materials that can adapt to fluctuating environments or execute many functions concurrently [25]. For example, composites integrating magnetic and fluorescent features are being engineered for use in bioimaging and biosensing, offering both diagnostic and therapeutic functionalities [26,27]. The potential of magnetic polymer composites resides in their capacity to tackle global issues in healthcare, energy, and environmental sustainability. In healthcare, these composites are anticipated to be essential in the advancement of personalized medicine, wherein therapies and technologies are customized to meet the specific needs of patients [28]. The incorporation of biodegradable and renewable polymers in the production of these composites is more prevalent, reflecting the heightened focus on sustainability and environmental accountability. Magnetic polymer composites are employed in robotics to fabricate soft, flexible actuators and sensors that replicate the movements and functionalities of biological tissues, facilitating the advancement of sophisticated prosthetics, wearable gadgets, and biomimetic robots [29]. In energy applications, these composites are being investigated for magnetic cooling systems and high-performance energy storage devices, where their lightweight and flexible characteristics provide considerable benefits compared to conventional materials.

Magnetic polymer conduits signify a significant leap in biomedical materials, providing a new platform for applications including tissue engineering and targeted therapy [30]. These conduits, generally tubular structures, are constructed by integrating magnetic nanoparticles into a biocompatible polymer matrix, including polylactic acid, chitosan, or silicone [31]. The resultant composite integrates the adaptability, tunability, and mechanical robustness of polymers with the magnetic responsiveness of nanoparticles, facilitating exact control via external magnetic fields. Magnetic polymer conduits are very beneficial in nerve and vascular regeneration, as they direct cell proliferation and tissue orientation through magnetic stimulation, enhancing healing results [32,33,34]. In medication delivery, they function as transporters for medicinal substances, facilitating targeted transport to specific locations, hence minimizing systemic side effects and improving treatment efficacy [35]. Moreover, their magnetic characteristics can be utilized in hyperthermia-based cancer treatments, wherein regulated heating eradicates tumor cells while preserving healthy tissues. These conduits are also utilized in minimally invasive operations, where their magnetic responsiveness enables accurate insertion or movement within the body. Recent improvements in fabrication techniques, like electrospinning and 3D printing, have facilitated the creation of conduits with regulated porosity, shape, and multifunctional attributes, thereby broadening their potential applications [36,37,38]. Notwithstanding these benefits, problems persist, such as attaining uniform nanoparticle dispersion, guaranteeing long-term biocompatibility, and scaling manufacturing for clinical applications [39]. Ongoing research is tackling these difficulties by focusing on the surface functionalization of nanoparticles, biodegradable polymers, and improved production techniques. Magnetic polymer conduits possess significant potential to revolutionize contemporary medicine by facilitating non-invasive, accurate, and multifunctional approaches to critical healthcare issues, especially in regenerative medicine, targeted drug delivery, and sophisticated therapeutic technologies [40].

This review article aims to deliver a thorough and current understanding of magnetic polymeric conduits and their transformational potential in biomedical applications, specifically in tissue engineering, regenerative medicine, and targeted therapeutics. The integration of polymers with magnetic nanoparticles imparts unique characteristics to these conduits, including magnetic responsiveness, biocompatibility, and mechanical flexibility, facilitating accurate and minimally invasive therapeutic treatments [41]. This review examines the essential characteristics of these materials, encompassing the varieties of polymers and magnetic nanoparticles employed in production methods and their multifunctional uses. Additionally, it tackles significant issues, including nanoparticle dispersion, biocompatibility, and long-term stability, while providing perspectives on emerging trends and future trajectories for the advancement of this domain.

## 2. Fundamentals of Magnetic Polymeric Conduits

Magnetic polymeric conduits are advanced composite materials that combine the functional benefits of polymers and magnetic substances for many biomedical applications. The design of these conduits commences with the meticulous selection of polymers, which function as the matrix material. Natural and manmade polymers are utilized based on the specific application requirements. Natural polymers, including chitosan, alginate, and collagen, are preferred due to their biocompatibility, biodegradability, and capacity to enhance cell adhesion and proliferation, rendering them suitable for tissue engineering and regenerative medicine. Synthetic polymers such as polylactic acid (PLA), polyethylene glycol (PEG), and polydimethylsiloxane (PDMS) provide superior mechanical qualities, chemical stability, and tunability, essential for applications demanding durability and prolonged usefulness [42,43,44,45]. The selection of polymer frequently relies on aspects including degradation rate, mechanical strength, and interaction with biological systems. The magnetic properties of these conduits are attained by integrating magnetic elements, usually as nanoparticles. Iron oxide nanoparticles are predominantly utilized for their biocompatibility, chemical stability, and superparamagnetic properties, which reduce residual magnetization following the cessation of an external magnetic field [46,47]. Alternative magnetic materials, such as cobalt and nickel, have superior magnetic strengths; nevertheless, their utilization in biomedical applications is limited due to toxicity and biocompatibility problems. Recent studies have investigated rare-earth magnetic nanoparticles, including neodymium–iron–boron (NdFeB), for specific applications necessitating robust magnetic fields. The magnetic particles endow the conduits with characteristics including reactivity to external magnetic fields, heating capabilities for hyperthermia therapy, and the capacity to direct or align tissues and cells during regeneration processes [48].

The incorporation of magnetic components into polymeric conduits employs many production techniques, each designed to ensure uniform dispersion and preserve the functional characteristics of both the polymer and the magnetic nanoparticles (Table 1). Solution blending is a straightforward and widely employed technique in which magnetic nanoparticles are combined with a polymer solution, subsequently undergoing solvent evaporation to yield the composite. Melt blending, a process that entails melting the polymer and nanoparticles to create a uniform mixture, is frequently utilized for thermoplastic polymers. In situ polymerization is a process in which magnetic nanoparticles are placed in a monomer solution, and polymerization is initiated to incorporate the particles into the developing polymer network. Advanced fabrication techniques, like electrospinning and 3D printing, are employed for intricate conduit designs. Electrospinning facilitates the fabrication of magnetic polymeric conduits featuring nanoscale fiber topologies, hence improving their mechanical properties and emulating the extracellular matrix for tissue engineering applications. Simultaneously, 3D printing provides exact control over the geometry and porosity of conduits, facilitating patient-specific designs and the integration of multifunctional attributes. The modification of magnetic nanoparticles’ surfaces, utilizing surfactants or polymers, is frequently implemented to optimize their dispersion in the polymer matrix and improve compatibility.

## 3. Fabrication Techniques

The manufacturing methods of polymeric conduits, particularly magnetic polymeric conduits, are essential for maintaining their structural integrity, functional efficacy, and medicinal relevance. These techniques entail the integration of magnetic nanoparticles, including iron oxide or cobalt ferrite, into polymer matrices such as polylactic acid, chitosan, or polydimethylsiloxane [79]. Techniques such as solution mixing, melt blending, and in situ polymerization are commonly employed to attain a uniform composite structure. Advanced methodologies, including electrospinning and 3D printing, have transformed the production of magnetic polymeric conduits by facilitating meticulous regulation of their shape, porosity, and alignment [80]. These technologies enable the fabrication of conduits with customized magnetic, mechanical, and biological characteristics, ensuring their appropriateness for applications in targeted drug delivery, tissue engineering, and regenerative medicine [81].

**Figure 1:**
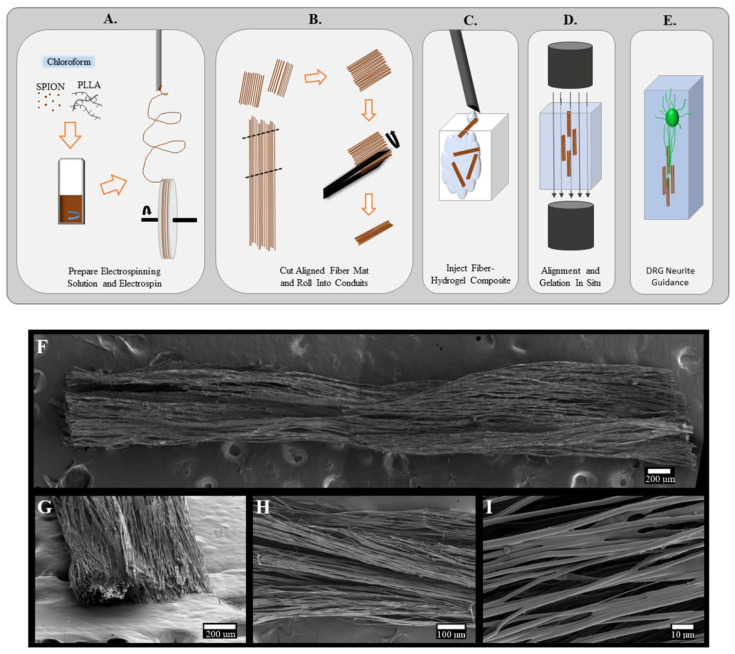
(**A**) Oleic acid-coated SPIONs are mixed with 8% PLLA in chloroform, followed by electrospinning onto a rotating mandrel to create aligned fibers. (**B**) Fiber mats are cut into 3 mm × 5 mm segments and rolled into small conduits. (**C**) Conduits and hydrogel are injected into a chamber. (**D**) A magnetic field is applied to align fibers within the hydrogel until solidified. (**E**) Fibers remain aligned after the field is removed, supporting neurite guidance from the dorsal root ganglion. (**F**–**I**) SEM images of a 6% SPION fiber conduit. Reproduced with permission from ref. [82]. © American Chemical Society.

Magnetic electrospun fibers are pertinent for minimally invasive biomaterial applications that aim to facilitate cell guiding. Magnetic electrospun fibers can be injected and subsequently magnetically positioned in situ, while the aligned fiber scaffolds offer uniform topographical guidance to cells [82]. Minor channels of oriented magnetic fibers were readily introduced into a collagen or fibrinogen hydrogel solution and realigned utilizing an external magnetic field. The aligned magnetic fibers provide internal directional guidance to neurites within a three-dimensional hydrogel model composed of collagen or fibrin, enhanced with Matrigel. A comprehensive schematic is provided in Figure 1 to illustrate the techniques employed in the fabrication of injectable fiber scaffolds (Figure 1A–E). The scanning electron microscopy (SEM) pictures depict the scaffold’s length, its edge, and the aligned fibrous architecture of the scaffolds (Figure 1F–I). Electrospun fibers were fabricated utilizing a base solution of 8 wt % poly(L-lactide) (PLLA) in chloroform. Superparamagnetic iron oxide nanoparticles (SPIONs) were integrated at concentrations of 2%, 4%, 6%, and 8% of the weight of PLLA.

A high-frequency magnetic field (MF) induces an electric current by energizing conductors, facilitating the induction of many biological processes, including alterations in cell fate and programming. This study demonstrates that electromagnetic carbon porous nanocookies (NCs) subjected to magnetic field treatment enhance magnetoelectric conversion, promoting growth factor release and cellular stimulation to drive neuronal cell differentiation and proliferation both in vitro and in vivo. The integration of four-dimensional printing technology exposes the NCs on the surface, thus enhancing cell adherence and facilitating the direct manipulation of electromagnetic stimulation of the cells. Significantly, substantial quantities of growth factor enclosed in NC@conduit yielded exceptional permeability and controlled release, enhancing the in vivo layers of myelin sheaths and guiding axon orientation one month post-implantation [83]. Nerve guide conduits (NGCs) have been extensively investigated as advanced options for nerve autografts and allografts for the treatment of peripheral nerve injuries. Nonetheless, the repair efficiency of NGCs requires substantial enhancement [84]. Functional NGCs that create a more advantageous milieu for facilitating axonal elongation and myelination are highly significant. In recent years, 3D printing technologies have been extensively utilized in the production of personalized and intricate structures, demonstrating significant potential for tissue engineering applications, particularly in the development of functioning NGCs. The capacity to capture, modify, and release individual cells on a surface is crucial for both fundamental investigations of cellular mechanisms and the advancement of innovative lab-on-chip miniaturized devices for biological and medicinal purposes. In a separate study, the authors described the utilization of magnetic domain barriers created in micro and nanostructures on a chip surface to manipulate individual yeast cells tagged with magnetic beads. We demonstrate that the suggested method preserves the viability of microorganisms, evidenced by the observation of tagged yeast cell division confined by domain walls throughout a 16 h period [85]. Three-dimensional (3D) nanomagnetic devices are garnering considerable attention owing to their prospective uses in computing, sensing, and biology. Nonetheless, their deployment encounters significant hurdles related to the production and characterization of 3D nanostructures [86]. A separate study introduced a technique for accurate on-chip modification of individual protein-coated magnetic beads utilizing geometrically limited magnetic domain walls (DWs) within nanowires. Through the regulation of nucleation, migration, and elimination of domain walls, the researchers exhibit the capacity to collect, transport, and release magnetic nanoparticles with nanometer-scale precision. This technology presents considerable potential for lab-on-chip applications, such as single-molecule investigations and biomagnetic sensing, by facilitating programmable circuits for molecular manipulation with ongoing process regulation [87]. Another study presented a thorough assessment of diverse techniques employed to fabricate nerve guidance conduits (NGCs) intended to promote nerve regeneration. The principal fabrication techniques examined encompass dip coating, solvent casting, micropatterning, electrospinning, and additive manufacturing. The merits and limits of each method are analyzed, along with solutions to address these issues. The review highlights the significance of conduit design and material selection in improving the efficacy of NGCs for nerve restoration applications [88].

Magnetic polymeric conduits are adaptable materials that merge the mechanical flexibility of polymers with the magnetic responsiveness of nanoparticles, rendering them very appropriate for diverse biological applications, including drug delivery, tissue engineering, and nerve regeneration. Coating and functionalization techniques are essential for improving the surface characteristics and functionality of these conduits to satisfy certain biomedical requirements. Surface coatings are extensively utilized to enhance biocompatibility and reduce immunological reactions. Natural polymers such as collagen, chitosan, and hyaluronic acid are frequently utilized to replicate the extracellular matrix, facilitating cell adhesion and proliferation, whereas synthetic polymers like polyethylene glycol (PEG) are employed as antifouling coatings to inhibit protein adsorption and mitigate inflammatory responses [89,90]. Methods including dip-coating, layer-by-layer assembly, and electrospinning guarantee the consistent application of these coatings. The incorporation of bioactive compounds significantly amplifies the therapeutic efficacy of magnetic polymeric conduits. Growth factors, including nerve growth factor (NGF) and vascular endothelial growth factor (VEGF), are affixed to the conduit surface via chemical conjugation techniques such as carbodiimide crosslinking to elicit biological responses. Moreover, peptide functionalization employing cell-adhesion patterns such as RGD (arginine-glycine-aspartic acid) promotes targeted cellular contacts, hence augmenting the regeneration potential of the conduits. Coating and functionalization techniques are crucial for customizing the physicochemical and biological features of magnetic polymeric conduits, facilitating their efficient application in modern biomedical fields.

The 3D printing of magnetic conduits has become a revolutionary method in biomedical engineering and other domains, necessitating meticulous regulation of magnetic characteristics and geometries. Researchers can construct conduits with customized magnetic functions by incorporating magnetic materials such as ferrites, iron oxide nanoparticles, or magnetically sensitive polymer composites into the printing process [91]. These conduits are very advantageous in applications, including nerve regeneration, medication administration, and targeted therapeutic systems [92]. In nerve regeneration, magnetic conduits serve as physical guides, and their magnetic qualities facilitate the alignment and stimulation of neuronal growth when subjected to an external magnetic field [93]. This dual functionality expedites healing by improving cellular organization and communication. In drug delivery, magnetic responsiveness facilitates precise spatial control, ensuring that medicinal medicines are administered precisely where required, hence avoiding side effects. Challenges encompass attaining a homogeneous distribution of magnetic elements inside the matrix and preserving the equilibrium between mechanical integrity and magnetic responsiveness. Nonetheless, progress in material science and printing methodologies is mitigating these constraints, thereby realizing the complete potential of magnetic conduits. Nerve guide conduits (NGCs) have been extensively investigated as advanced options for nerve autografts and allografts for the treatment of peripheral nerve damage. Nonetheless, the repair efficiency of NGCs requires substantial enhancement. Functional NGCs that create a more advantageous milieu for facilitating axonal elongation and myelination are highly significant. NGCs can be fabricated incrementally using rapid prototyping machines facilitated by CAD technology that captures and digitizes the intricate microarchitectural data of native tissue from images obtained through computed tomography (CT) or magnetic resonance imaging (MRI) [94]. Jakus et al. showed a three-dimensional printable graphene (3DG) composite composed predominantly of graphene and secondarily of polylactide-co-glycolide, a biocompatible elastomer, which was 3D-printed from a liquid ink. This ink can be employed under ambient circumstances by extrusion-based 3D printing to fabricate graphene structures with features as small as 100 μm, consisting of as few as two layers (objects < 300 μm thick) or several layers (objects >10 cm thick) [95]. The resultant 3DG material exhibits mechanical robustness and flexibility while maintaining electrical conductivities over 800 S/m, representing an order of magnitude enhancement compared to previously documented 3D-printed carbon materials. In vitro tests conducted in a basic growth medium, devoid of neurogenic cues, demonstrate that 3DG enhances human mesenchymal stem cell (hMSC) adhesion, viability, proliferation, and neurogenic differentiation, accompanied by considerable overexpression of glial and neuronal genes. This corresponds with hMSCs assuming very elongated morphologies resembling axons and presynaptic terminals. In vivo studies demonstrate that 3DG exhibits favorable biocompatibility for a duration of at least 30 days. Tissue-engineered conduits hold significant potential for bridging peripheral nerve deficits by offering physical guidance and biological signals. A versatile approach for incorporating support cells into a conduit with specified designs is required [96]. A 3D-printing method is utilized to fabricate a bio-conduit with engineered components for peripheral nerve regeneration. This bio-conduit comprises a cryopolymerized gelatin methacryloyl (cryoGelMA) gel populated with adipose-derived stem cells (ASCs). Utilizing 3D-printed “lock and key” molds, the cryoGelMA gel is configured into conduits featuring various shapes, including the engineered multichannel, bifurcating, and customized structures.

## 4. Biomedical Applications

### 4.1. Drug Delivery

**Figure 2:**
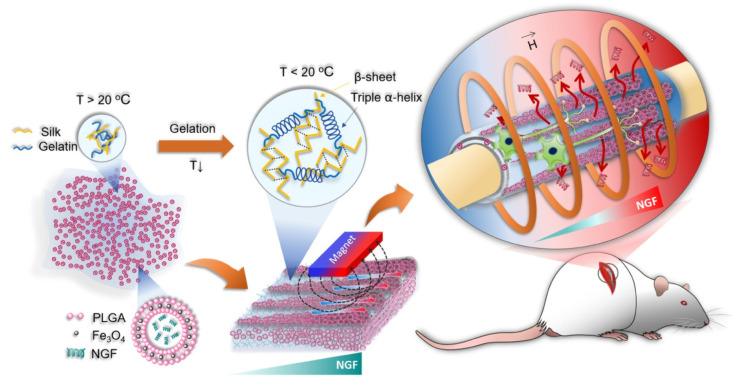
Schematic representation of the fabrication of an NGF@MPs-GS nerve conduit. Reproduced with permission of ref. [101]. © American Chemical Society.

Drug delivery is a vital domain of biomedical research focused on enhancing the accuracy, effectiveness, and safety of therapeutic interventions. Magnetic conduits present a promising platform for targeted drug delivery because of their distinctive capacity to react to external magnetic fields [97,98]. Embedding magnetic nanoparticles or magnetically responsive materials into biocompatible conduits facilitates regulated and localized drug release, hence reducing systemic side effects. This feature is especially beneficial for addressing illnesses necessitating targeted therapeutic intervention, such as cancer, infections, or localized inflammation [99]. The multifunctionality of magnetic conduits, particularly their capacity for real-time imaging and monitoring, augments their efficacy in drug administration, rendering them an invaluable asset for the advancement of customized medicine [100]. A gelatin/silk (GS) hydrogel was utilized to create a nerve guidance conduit (NGC) due to its intrinsic reversible thermoresponsive sol-to-gel phase transformation capability, which enabled the swift three-dimensional (3D) micropatterning of the embedded nerve growth factor (NGF)-loaded magnetic poly(lactic-co-glycolic acid) (PLGA) microcapsules, referred to as NGF@MPs, through multiple magnetic guidance techniques (Figure 2). The thermally adjustable viscosity of GS facilitated the fast creation of a 3D gradient and a linear arrangement of NGF@MPs, resulting in magnetically regulated 3D gradient release of NGF to improve topographical nerve guidance and wound healing in peripheral nerve injuries. The micropatterned hydrogel, designated NGF@MPs-GS, exhibited a corrugated topography with a pattern height H of 15 μm, leading to a linear axon alignment in over 90% of the cells. Furthermore, an external magnetic field facilitated spatiotemporal control of NGF release, enabling neurite elongation that was nearly twice as long as that observed in the group receiving external NGF supplementation.

**Figure 3:**
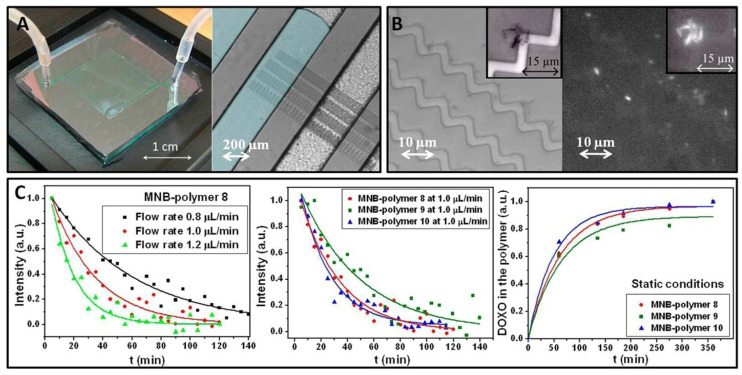
(**A**) A typical experiment chip (left panel) and microfluidic channels with zigzag-shaped permalloy (Ni80Fe20) microstripes (right panel). The serpentine’s empty half is light gray, while the left panel’s liquid-filled channel is light blue. (**B**) Bright-field (BF) image of MNB-polymer 8 aggregates trapped by DWs at several corners of one of the zigzag-shaped magnetic conduits at 45 °C and FITC-filtered image showing the aggregates’ fluorescence due to doxorubicin molecules in the magnetic nanobeads. In the BF and FITC filtered image, the insets zoom in on a trapped polymeric aggregate. (**C**) Fluorescence intensity vs. time curves of MNB-polymer 8 at various flow rates (left panel); MNB-polymer 8, 9, and 10 at 1.0 μL/min flow rate (central panel); and cumulative DOXO release profile from MNBs coated with polymers 8, 9, and 10 without flow rate (right panel). Reproduced with permission of ref. [103]. © American Chemical Society.

Magnetic fiber composites that integrate superparamagnetic iron oxide nanoparticles (SPIONs) with electrospun fibers exhibit potential in tissue engineering applications. The regulated attachment of SPIONs to the fibers during electrospinning produces biocompatible magnetic composites while preserving the intended fiber shape. Funnell et al. evaluated the efficacy of SPION-grafted scaffolds in conjunction with magnetic fields to enhance neurite outgrowth by offering contact guiding through aligned fibers and mechanical stimulation via SPIONs in the magnetic field. Neurite outgrowth from primary rat dorsal root ganglia (DRG) was evaluated using explants cultured on aligned control and SPION-grafted electrospun fibers, as well as on non-grafted fibers with SPIONs distributed in the culture medium. To ascertain the ideal magnetic field stimulation for enhancing neurite outgrowth, we created a static, alternating, and linearly moving magnet, thereafter simulating the magnetic flux density throughout various regions of the scaffold over time [102]. Leal et al. described a method for cultivating thermo-responsive polymer shells on the surfaces of magnetic nanocarriers composed of numerous iron oxide superparamagnetic nanoparticles contained within poly(maleic anhydride-alt-1-octadecene) polymer nanobeads [103]. Tunable phase transition temperatures between 26 and 47 °C under physiological conditions can be attained, contingent upon the comonomers and their relative composition. They demonstrated that thermo-responsive nanobeads are effective for targeted drug delivery through a microfluidic platform that integrates magnetic nanostructures and channels that replicate the capillaries of the circulatory system (Figure 3), utilizing both thermal and magnetic activation.

### 4.2. Magnetic Hyperthermia

Magnetic polymeric conduits have developed as effective instruments for targeted cancer treatment, especially via a method called magnetic hyperthermia. This method employs the distinctive characteristics of magnetic nanoparticles (MNPs), which produce localized heat when exposed to an alternating magnetic field. The incorporation of these MNPs into polymeric conduits facilitates targeted and regulated heating, permitting accurate therapy of malignant regions while reducing harm to adjacent healthy cells. The conduits, often configured as implants or delivery systems, are carefully positioned adjacent to or within the tumor site, facilitating the release of MNPs in close proximity to cancer cells. Magnetic hyperthermia (MH, or magnetic fluid hyperthermia) is a process wherein magnetic energy is converted into thermal energy via the rapid oscillations of magnetic nanoparticles induced by an alternating magnetic field (AMF). When subjected to an alternating magnetic field, the MNPs undergo rapid oscillations, generating localized heat that elevates the temperature of adjacent tissue to a degree capable of causing cell death, either via direct thermal injury or by stimulating cellular stress responses that augment the effectiveness of chemotherapy or immunotherapy. The utilization of magnetic polymeric conduits presents multiple benefits, such as non-invasive therapy, targeted energy delivery to the tumor, and diminished systemic adverse effects. The polymeric materials utilized in these conduits are frequently biocompatible and biodegradable and can be customized to improve medication delivery efficacy in conjunction with magnetic heating. Moreover, these conduits can be 3D printed or fabricated to possess intricate geometries, allowing for design flexibility and ensuring that the treatment can be tailored to individual patients. The capacity to accurately regulate heat generation in real time creates opportunities for adaptive and responsive treatment methodologies. With ongoing research, magnetic polymeric conduits are set to play a crucial role in the future of cancer therapy, providing a localized, focused, and efficient method for tumor treatment.

**Figure 4:**
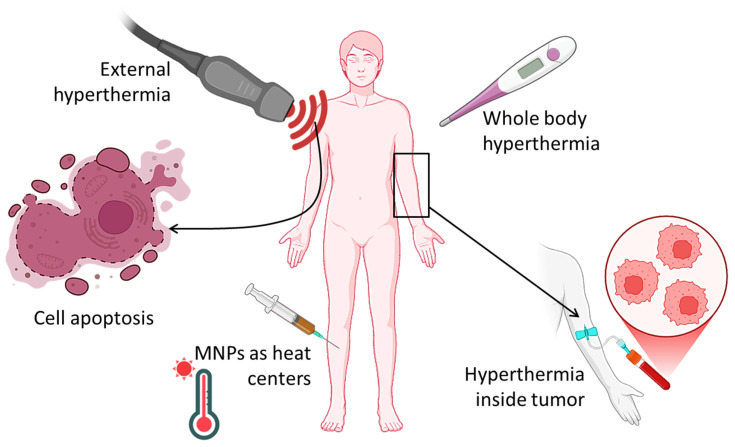
Types of hyperthermia and action in the human body.

Despite the application of hyperthermia (HT) in clinical cancer treatment, maintaining localized temperature control remains a challenge. As of now, three primary forms of HT have been utilized in clinical practice (Figure 4): whole-body, regional, and local. Whole-body hyperthermia, commonly utilized as an adjunct for metastatic disease, is administered via heated blankets and thermal chambers. Regional hyperthermic therapy entails the infusion of hot fluids containing anticancer agents into the peritoneal cavity. Nonetheless, the therapeutic utilization of both whole-body and regional hyperthermia is constrained by significant adverse effects, particularly gastrointestinal symptoms (diarrhea, nausea, and vomiting) and cardiac consequences (thrombosis, myocardial ischemia, and perhaps heart failure).

**Figure 5:**
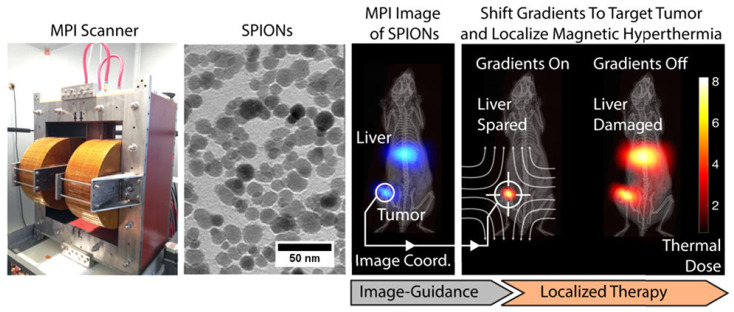
Gradient fields enabling arbitrary magnetic hyperthermia therapy localization in vivo using magnetic particle imaging. Reproduced with permission from ref. [104]. © American Chemical Society.

Image-guided cancer treatment allows clinicians to accurately locate and address malignancies. We present in vivo results demonstrating that the novel imaging technique, magnetic particle imaging (MPI), may be integrated with magnetic hyperthermia to provide an image-guided theranostic platform. Magnetic particle imaging (MPI) is a non-invasive three-dimensional tomographic imaging technique characterized by excellent sensitivity and contrast, absence of ionizing radiation, and linear quantitativeness at any depth without view restrictions [104]. The identical superparamagnetic iron oxide nanoparticle (SPION) tracers visualized in magnetic particle imaging (MPI) can also be stimulated to produce heat for magnetic hyperthermia. This study presents a theranostic platform that utilizes quantitative MPI imaging for treatment planning and employs MPI gradients for the spatial localization of magnetic hyperthermia in specifically chosen areas (Figure 5). This tackles a significant issue of traditional magnetic hyperthermia—SPIONs administered systemically aggregate in off-target organs (e.g., liver and spleen), and the challenge of localizing hyperthermia leads to unintended thermal injury to these organs.

The technique of signal localization via gradient fields in MPI can similarly facilitate the spatial localization of magnetic fluid hyperthermia (MFH), enabling targeted heating within the body and offering enhanced control and flexibility in MFH applications. Moreover, MPI and MFH can be combined into a singular device for concurrent MPI–MFH functionality and effortless transition between imaging and therapeutic modes. This study presents computational and experimental work that quantifies the degree of spatial localization of magnetic fluid heating (MFH) via MPI systems [105].

### 4.3. Tissue Engineering

**Figure 6:**
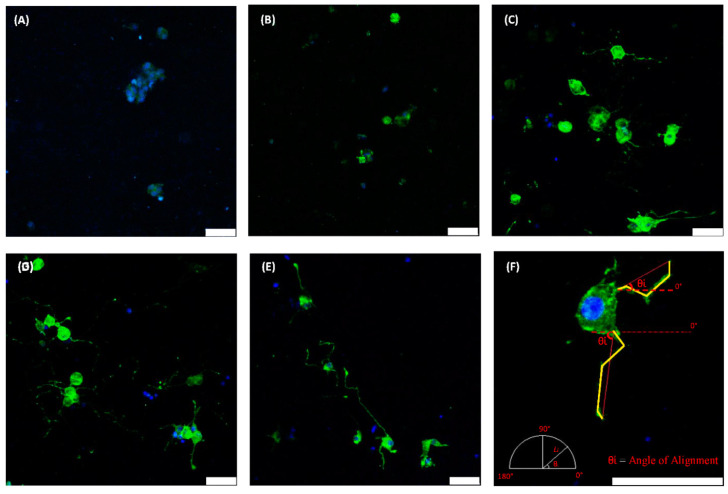
Confocal images of Phalloidin/DAPI staining of PC12 cells in plain collagen scaffolds (**A**), GMNP-incorporated scaffolds without magnetic field treatment (**B**), “magnetic particle string” scaffolds (**C**), CMP-functionalized scaffolds (**D**), and “magnetic anchor” scaffolds (**E**). Image (**F**) shows how differentiated PC12 cells were measured; yellow lines show axonal length, and red lines demonstrate alignment. The angle of alignment (θ) is determined from the horizontal plane (0°). Reproduced with permission from ref. [115]. © MDPI.

Magnetic stimulation, a non-invasive therapy, utilizes magnetic nanoparticles. Gold and iron oxide magnetic nanoparticles are distributed throughout mediums such as hydrogels and scaffolds via crosslinking or in situ synthesis [106,107,108,109,110]. Tissue engineering research is undergoing a significant transformation toward cell culture on biomimetic materials [93] that emulate the characteristics of genuine tissues from where the cells originate. Limited research has been conducted in this area concerning neural cells, especially within the context of nanomedicine [107]. Platforms like MNPs have demonstrated efficacy as multipurpose instruments for cell tracking and genetic modification of brain transplant populations. A cohort of researchers conducted the inaugural assessment of magnetoreception technology, which enhances magnetic nanoparticle delivery through applied magnetic fields, demonstrating considerable therapeutic potential, and its application in the genetic modification of neural stem cells, a clinically significant population cultivated in biomimetic hydrogels [78,111,112,113]. We illustrate that the safe deployment of a magnetic field significantly improves MNP-mediated transfection of neural stem cells (NSCs) cultivated as 3D spheroid structures in collagen, which more accurately mimics the inherent mechanical and structural characteristics of neural tissue compared to conventional rigid substrates. Additionally, recognizing that MNP uptake is facilitated by endocytosis, we examined the membrane activity of NSCs cultured on both soft and hard substrates. Utilizing high-resolution scanning electron microscopy, we demonstrated that neural stem cells exhibit reduced membrane activity on soft substrates in comparison to hard ones, a discovery that may significantly influence MNP-mediated cellular engineering strategies in physiologically relevant systems [114]. The deployment of neural scaffolds featuring a precisely defined microarchitecture, produced using conventional methods like electrospinning and microfluidic spinning, necessitates surgical intervention at the damage site. To mitigate the risks associated with aciurgy, novel treatment techniques are being pursued. This has resulted in a surge of research on injectable hydrogels in recent years. Nonetheless, limited research has been undertaken to regulate the constituents of these injectable hydrogels to create analogous scaffolds with a precisely specified microarchitecture. Methods such as “magnetic particle string” and biomimetic amphiphile self-assembly are now employed to accomplish this objective. We devised a “magnetic anchor” technique to enhance the alignment of collagen fibers in injectable 3D scaffolds. This approach employs GMNP (gold magnetic nanoparticles) “anchors” coated with CMPs (collagen mimetic peptides) that “link” them to collagen fibers. The use of a magnetic field during the gelling process aligns the collagen fibers properly (Figure 6). A study showed that the utilization of CMP-functionalized GMNPs in a magnetic field markedly enhances the alignment of collagen fibers, hence improving the orientation of rat pheochromocytoma cell line (PC12) neurites [115]. The elongation of these neurite extensions, which were demonstrated to be considerably longer, was also enhanced. The PC12 cultured in collagen scaffolds constructed with the “magnetic anchor” technique exhibit cellular vitality comparable to that of untreated collagen scaffolds.

**Figure 7:**
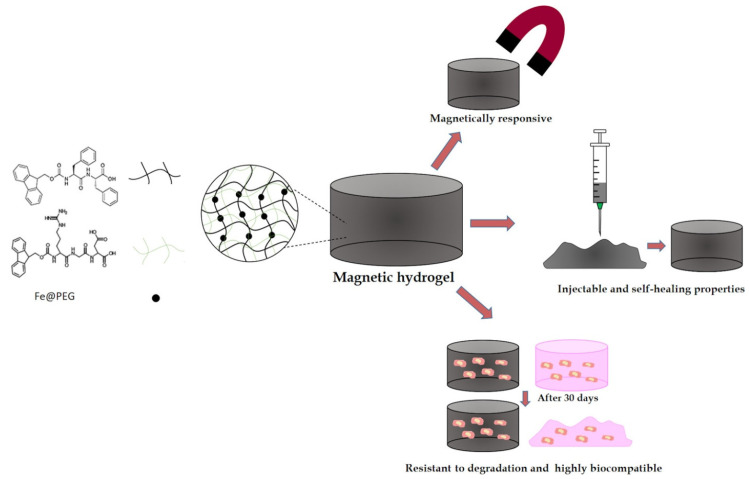
Diagram illustrating the characteristics of short-peptide supramolecular magnetic hydrogels. Reproduced with permission from ref. [116]. © American Chemical Society.

Embedded MNP enables the remote and on-demand modulation of the hydrogel’s physical properties through the application of an external magnetic field. Furthermore, they provide enduring modifications in the mechanical characteristics of the hydrogel, together with changes in the micro and macro-porosity of its 3D architecture, hence possessing the potential to induce anisotropy (Figure 7). This study meticulously examined the properties of biocompatible and biodegradable hydrogels composed of Fmoc-diphenylalanine (Fmoc-FF) and Fmoc-arginine-glycine-aspartic acid (Fmoc-RGD) short peptides, into which magnetic nanoparticles (MNP) were integrated, utilizing physicochemical, mechanical, and biological methodologies.

Huang et al. employed a magnetic field engineered to generate mechanical stimulation for mesenchymal stem cells, thereby enhancing chondrogenic development. Gelatin, β-cyclodextrin (β-CD), and magnetic nanoparticles (Fe_3_O_4_) were integrated to create a magnetic nanocomposite hydrogel for internal response applications. Their findings demonstrated the enhanced mechanical stimulation of MSCs when combined with a magnetic nanocomposite hydrogel. They exhibit improved chondrogenic differentiation and facilitate cartilage healing in the presence of a magnetic field. This study introduces a novel form of magnetic tissue-engineered cartilage and a potential approach for utilizing magnetic stimuli to facilitate the repair of articular cartilage lesions in rabbit knees [117]. To repair soft aligned tissues in living creatures, minimally invasive biomaterials are necessary to construct 3D microenvironments that replicate the native architecture of the tissue. An adjustable injectable hydrogel has been disclosed, enabling accurate in situ engineering of the construct’s anisotropy [118]. This substance is characterized as an Anisogel, signifying an innovative form of tissue regenerative therapy. The Anisogel consists of a pliable hydrogel that encases magneto-responsive, cell-adhesive short fibers, which align in situ according to a low external magnetic field prior to the complete gelation of the matrix. The magnetic field can be eliminated following the gelation of the biocompatible gel precursor, which stabilizes the aligned fibers and maintains the anisotropic structure of the Anisogel. Fibroblasts and nerve cells exhibit unidirectional growth and extension within Anisogels, unlike hydrogels devoid of fibers or those containing randomly oriented fibers. The neurons within the Anisogel exhibit spontaneous electrical activity, with calcium signals disseminating down the material’s anisotropy axis. Tissue engineering research is undergoing a significant paradigm shift toward cell culture on biomimetic materials that emulate the characteristics of native tissues from where the cells originate. Limited research has been conducted in this area concerning neural cells, especially within the realm of nanomedicine [119]. Hydrogel scaffolds are notably significant for tissue engineering applications due to their capacity to establish a conducive environment that simulates in vivo settings. Nonetheless, the hierarchically organized anisotropic structure seen in numerous native tissues and cellular components is challenging to replicate in 3D scaffolds [120]. A different group documented the integration of magnetic nanoparticle-decorated reduced graphene oxide (m-rGO) into a collagen hydrogel. The magneto-responsive m-rGO was oriented within the collagen hydrogel during gelation with the introduction of a low external magnetic field. This nanocomposite hydrogel, featuring magnetically aligned m-rGO flakes, effectively encapsulates neuroblastoma cells (SH-SY5Y), facilitating cell differentiation and boosting directed cell growth because of its superior biocompatibility and electrical conductivity. The directionally oriented and differentiated SH-SY5Y cells in the m-rGO collagen hydrogel exhibited the propagation of calcium signals along the orientation axis. This technique can be utilized for the development of magnetically responsive materials with potential biomedical applications.

### 4.4. Magnetically Guided Conduits

Magnetically guided conduits are a novel category of biomedical devices engineered to utilize magnetic fields for improved control and accuracy in several therapeutic applications. These conduits, typically composed of polymer-based structures infused with magnetic particles, function as channels for directing therapeutic materials, including medications or stem cells, to targeted locations within the body. The capacity to manipulate the flow of these conduits using external magnetic fields markedly enhances the accuracy of targeted treatments, diminishing the likelihood of side effects and augmenting the effectiveness of therapies. Magnetically directed conduits facilitate the accurate delivery of therapeutic molecules to diseased tissues, especially in difficult regions such as deep tissues or tumors. Magnetic fields enable real-time, non-invasive manipulation of the conduit’s trajectory, enabling precise delivery of the therapeutic substance, hence reducing systemic distribution and enhancing medicinal efficacy. These conduits are under investigation for use in tissue engineering and regenerative medicine. Magnetic nanoparticles included in biodegradable polymers can direct the migration of stem cells or growth hormones, thereby enhancing tissue regeneration at targeted sites. The capacity to regulate these channels presents significant potential for enhancing the efficacy of tissue repair, particularly in intricate injuries or disorders necessitating highly localized intervention.

Through the application of an external magnetic field, spatiotemporal control over NGF release was achieved, resulting in neurite elongation nearly double that observed in the group receiving external NGF supplementation. A prototype of the nerve guidance conduit (NGC) was ultimately constructed and implanted into the damaged sciatic nerve. The patterned implant, aided by magnetic stimulation, exhibited expedited recovery of motor function within 14 days post-implantation. It additionally facilitated the improvement of axon outgrowth and remyelination after 28 days. This NGC, featuring adjustable mechanical, pharmacological, and topographical stimuli, is a promising platform for the improvement of neuron regeneration [101]. The preliminary provision of small-scale magnetic devices, including microrobots, is a crucial yet frequently neglected component for their use in therapeutic applications. The implementation of these devices in the dynamic milieu of the human body poses considerable hurdles due to their distribution influenced by circulatory currents. A method is presented for the efficient delivery of a swarm of magnetic nanoparticles in fluidic flows [121]. The microcatheter and reservoir are designed to facilitate magnetic steering and the injection of magnetic nanoparticles. Experiments were performed to illustrate this strategy, employing a spinal cord phantom that simulates intrathecal catheter delivery for central nervous system applications. The results indicate that the proposed microcatheter effectively concentrates nanoparticles at the targeted site by precisely manipulating magnetic field gradients, presenting a viable solution for the controlled deployment of untethered magnetic micro-/nano-devices within the intricate physiological circulatory systems of the human body [122]. Hu et al. reported on the utilization and advancement of two magnetically directed porogen assembly techniques employing magnetic sugar particles (MSPs) for scaffold manufacturing. A patterning device is employed to align MSPs according to specified templates [123]. A magnetic sheet film is produced by combining poly(vinyl alcohol) (PVA) with NdFeB powder to direct the MSPs. Following the casting of poly(l-lactide-co-ε-caprolactone), PLCL, and the extraction of the sugar template, a scaffold with spherical pores is produced. Directionally aligned polycaprolactone/triiron tetraoxide (PCL/Fe_3_O_4_) fiber scaffolds were initially fabricated using the electrospinning process and subsequently grafted with IKVAV peptide to modulate dorsal root ganglion (DRG) growth and axon extension in peripheral nerve regeneration. The findings indicated that oriented, aligned magnetic PCL/Fe_3_O_4_ composite scaffolds were effectively fabricated using the electrospinning approach and exhibited favorable mechanical properties and magnetic responsiveness [124]. The PCL/Fe_3_O_4_ scaffolds with varying Fe_3_O_4_ concentrations exhibited no cytotoxicity, demonstrating their excellent biocompatibility and little cytotoxic effects. The IKVAV-functionalized PCL/Fe_3_O_4_ scaffolds effectively directed and enhanced the axial extension of axons, whereas the application of an external magnetic field and the grafting of IKVAV peptides dramatically augmented the growth of dorsal root ganglia (DRGs) and axons. Tissue-engineered conduits hold significant potential for bridging peripheral nerve deficits by offering physical guidance and biological signals. A versatile approach for incorporating support cells into a conduit with specified designs is required. A 3D-printing method is utilized to fabricate a bio-conduit with engineered components for peripheral nerve regeneration [125]. This bio-conduit comprises a cryopolymerized gelatin methacryloyl (cryoGelMA) gel populated with adipose-derived stem cells (ASCs). The cryoGelMA gel is built into conduits with various shapes, including planned multichannel, bifurcating, and customized structures, by utilizing 3D-printed “lock and key” molds for modeling.

## 5. Biocompatibility and Toxicity

MNPs have become pivotal instruments in biomedical applications, including drug delivery, imaging, and hyperthermia, yet their clinical efficacy is closely linked to their biocompatibility and toxicity characteristics. The physicochemical characteristics of MNPs, such as dimensions, morphology, surface chemistry, and magnetic core composition, are crucial in influencing their interactions with biological systems. Particle size markedly affects biodistribution and clearance mechanisms—particles under 10 nm are swiftly eliminated via renal clearance, whereas those over 200 nm are mostly sequestered by the reticuloendothelial system (RES), frequently resulting in accumulation in the liver and spleen. The morphology of nanoparticles significantly influences cellular uptake; spherical nanoparticles are often ingested more effectively than rod- or cube-shaped counterparts, attributable to variations in cellular membrane wrapping dynamics. Surface chemistry is vital, as the functionalization with biocompatible polymers such as polyethylene glycol (PEG), dextran, or chitosan reduces nonspecific protein adsorption, immunogenicity, and reticuloendothelial system clearance, hence improving biocompatibility. Conversely, uncoated or highly reactive surfaces may provoke inflammation, oxidative stress, and cellular toxicity. The composition of the magnetic core influences toxicity; for example, iron oxide nanoparticles (IONPs) are typically biocompatible owing to their natural iron metabolism pathways, whereas cobalt- and manganese-based cores frequently demonstrate cytotoxicity due to the liberation of toxic ions such as Co^2+^ and Mn^2+^, which interfere with mitochondrial function and enzymatic pathways. Toxicity is frequently influenced by mechanisms including oxidative stress, inflammation, and DNA damage, in addition to intrinsic material qualities. Oxidative stress is notably critical, as the breakdown of MNPs in lysosomal compartments releases free iron ions, initiating Fenton chemistry and producing reactive oxygen species (ROS). Increased amounts of reactive oxygen species (ROS) can impair lipids, proteins, and nucleic acids, undermining cellular integrity and functionality. Although surface coatings and core–shell designs can mitigate these negative effects, prolonged in vivo studies indicate that MNP buildup in organs may result in persistent inflammation, fibrosis, or impaired organ function. Furthermore, the development of a protein corona upon contact with biological fluids might significantly modify the pharmacokinetics and immunogenicity of MNPs, highlighting the necessity of methodical surface engineering. Advancing the clinical translation of MNPs necessitates a comprehensive understanding of their interactions at the nano-bio interface, as well as the creation of customized surface chemistries and hybrid architectures to reduce toxicity while enhancing therapeutic and diagnostic effectiveness.

Recent improvements in magnetic conduits aim to tackle the issue of biocompatibility through surface functionalization and the incorporation of biocompatible materials. Surface functionalization with biopolymers such as polyethylene glycol (PEG) or natural compounds like collagen has demonstrated a reduction in cytotoxicity while promoting cell adhesion and proliferation [126]. Furthermore, bioactive coatings that enhance certain cellular interactions, such as RGD peptides, are being progressively utilized to augment the integration of magnetic conduits inside biological contexts. Moreover, the selection of biodegradable polymer matrices, like polylactic acid (PLA), polycaprolactone (PCL), or gelatin, has demonstrated efficacy in guaranteeing biocompatibility [127]. These compounds deteriorate over time into non-toxic metabolites, reducing prolonged inflammatory responses. These methods enhance the safety of magnetic conduits for in vivo applications, especially in nerve regeneration and medication delivery systems. Innovations in 3D printing and nanoparticle synthesis are addressing scalability concerns [128]. High-throughput 3D printing techniques, including multi-nozzle extrusion and DIW, enable the concurrent production of many conduits with uniform quality and accuracy. Improvements in material compositions, including the optimization of magnetic ink viscosity and flow characteristics, guarantee reliable and efficient extrusion in large-scale manufacturing. Scalable synthesis methods for magnetic materials, such as microwave-assisted techniques and green chemistry approaches, have enhanced manufacturing yields and decreased prices [129,130]. These approaches guarantee consistent particle size and magnetic characteristics, which are essential for preserving the operational efficacy of conduits.

## 6. Future Directions and Perspectives

The future of magnetic polymeric conduits lies in advancing their design and multifunctionality to meet the growing demands of biomedical applications, particularly in nerve regeneration, vascular repair, and targeted drug delivery. Emerging trends in conduit design are moving toward the incorporation of dynamic, adaptive architectures that can respond to external stimuli, such as magnetic fields, to achieve controlled mechanical, chemical, and biological functionality. For instance, the integration of programmable magnetic fields is expected to enable precise manipulation of conduit shape, alignment, and therapeutic payload release. Recent advances in additive manufacturing, including 3D and 4D printing, offer exciting possibilities to fabricate conduits with spatially defined magnetic properties, tailored geometries, and gradient structures that mimic native tissue environments. These developments are paving the way for more efficient nerve guidance channels and vascular conduits that facilitate directional cell migration and angiogenesis under applied magnetic stimuli.

Incorporating multifunctional properties into magnetic conduits represents another critical direction in this field. Beyond their magnetic responsiveness, conduits are being engineered to exhibit additional functionalities, such as electrical conductivity for neural stimulation or optical properties for real-time imaging and tracking. The incorporation of electrically conductive polymers, such as polypyrrole or graphene-based composites, into magnetic conduits can enhance neural signal transmission while maintaining biocompatibility. Similarly, the integration of optical functionality through fluorescent or plasmonic nanoparticles allows for non-invasive monitoring of conduit performance and tissue regeneration. Hybrid systems combining magnetic, electrical, and optical properties are poised to revolutionize regenerative medicine by enabling synergistic stimulation of tissue repair processes while offering precise diagnostic and therapeutic control.

Looking forward, the clinical translation of magnetic polymeric conduits will depend on addressing several regulatory and safety challenges. Standardized protocols for evaluating the long-term biocompatibility, biodegradability, and toxicity of these conduits under physiological conditions are urgently needed. Additionally, understanding the impact of chronic exposure to magnetic fields on human tissues and the degradation behavior of magnetic nanoparticles within the body remains a critical area of investigation. Regulatory frameworks will need to evolve to accommodate the complexity of multifunctional conduits, especially in terms of ensuring their reproducibility, sterility, and scalability for clinical use. As the field progresses, collaboration between material scientists, biomedical engineers, and clinicians will be essential to align the design and functionality of magnetic polymeric conduits with real-world clinical needs. These multidisciplinary efforts, combined with advancements in manufacturing and characterization techniques, hold immense potential to transform magnetic conduits from experimental prototypes into viable clinical solutions for regenerative medicine and beyond.

## 7. Conclusions

Magnetic polymeric conduits signify a groundbreaking advancement in regenerative medicine and biomedical engineering, merging the distinct benefits of magnetic nanoparticles with the adaptability of polymeric materials. This study underscores significant progress in the design, construction, and functionalization of these conduits, highlighting their capacity to facilitate tissue regeneration, improve drug delivery, and enable remote manipulation via magnetic fields. Notably, the incorporation of magnetic properties into polymeric matrices facilitates precise cellular behavior regulation, enhances scaffold alignment, and enables localized delivery of therapeutic agents. Looking forward, tissue engineering appears to be one of the most promising application areas for magnetic polymeric conduits. Future research could focus on developing scaffolds that mimic the native extracellular matrix more closely, incorporating features such as dynamic magnetic stimulation to enhance cell proliferation and differentiation. For example, advanced fabrication techniques like 4D printing could be explored to create conduits that adapt dynamically to the tissue environment. Additionally, integrating multifunctional capabilities, such as combining electrical conductivity for neural stimulation with optical tracking for real-time monitoring, could further enhance their clinical utility. While challenges remain, including long-term biocompatibility, biodegradability, and scalable fabrication, advances in material science and nanotechnology are rapidly addressing these issues. By overcoming these barriers, magnetic polymeric conduits hold immense potential to revolutionize tissue engineering and personalized regenerative medicine, offering targeted, efficient, and adaptive solutions to complex biomedical challenges.

## Figures and Tables

**Table 1:** Different types of magnetic polymeric systems.

Aspect	Details	Ref.
Types of Polymers	Natural polymers: Chitosan, alginate, collagen (biocompatible, biodegradable, supports cell adhesion and growth).	[49,50,51,52]
	Synthetic polymers: Polylactic acid (PLA), polyethylene glycol (PEG), polydimethylsiloxane (PDMS) (durable, stable, tunable mechanical properties).	[53,54,55]
Magnetic Materials Used	Iron oxide nanoparticles: Magnetite (Fe_3_O_4_), maghemite (γ-Fe_2_O_3_) (biocompatible, stable, superparamagnetic).	[56,57,58]
	Other magnetic materials: Cobalt, nickel (higher magnetic strength but potential toxicity issues).	[59]
	Rare-earth magnetic nanoparticles: Neodymium-iron-boron (NdFeB) (strong magnetic properties for specialized applications).	[60]
Integration Methods	Solution blending: Magnetic nanoparticles mixed with polymer solution, followed by solvent evaporation.	[1,4]
	Melt blending: Heating polymer and nanoparticles to form a homogeneous composite.	[1,61,62]
	In situ polymerization: Dispersing nanoparticles in monomers and initiating polymerization.	[7,63,64]
	Electrospinning: Produces nanoscale fibers with enhanced mechanical properties and extracellular matrix mimicry.	[65]
	Three-dimensional printing: Enables precise control over conduit geometry and porosity for patient-specific designs.	[66]
Surface Functionalization	Use of surfactants or polymers to improve nanoparticle dispersion and compatibility with the polymer matrix.	[67,68,69]
Key Applications	Regenerative medicine: Tissue scaffolds for cell alignment and regeneration.	[32,70,71,72]
	Targeted drug delivery: Magnetic field-guided delivery of therapeutic agents.	[73,74,75]
	Hyperthermia therapy: Controlled heating for cancer cell destruction.	[76,77,78]

## Data Availability

Data are available by reasonable request.
